# Upcycling Hospital Lab Polypropylene Waste into a Fully Integrated Additive Manufacturing Electroanalytical Sensing Platforms

**DOI:** 10.1021/acssusresmgt.5c00393

**Published:** 2025-11-03

**Authors:** Muhzamil A. Khan, Elena Bernalte, Danielle Stephens, Robert D. Crapnell, Craig E. Banks

**Affiliations:** 1 Faculty of Science and Engineering, 5289 Manchester Metropolitan University, Dalton Building, Chester Street, Manchester M1 5GD, Great Britain; 2 RecycleLab Ltd., 101 New Cavendish Street, London W1W 6XH, United Kingdom

**Keywords:** additive manufacturing, recycling, upcycling, sustainable development goals, plastic waste, polypropylene

## Abstract

Plastic waste is one of the largest contributors to landfill waste globally, with the healthcare industry contributing a large proportion of this. Recycling has been established as a key point of focus to reduce this waste, as addressed in The United Nations Sustainable Development Goals. To this end, this work demonstrates the upcycling of hospital lab waste poly­(propylene) (PP) into a new conductive filament for additive manufacturing, using a zero solvent methodology and incorporating 30 wt % carbon black as a conductive filler. The filament showed excellent low-temperature flexibility, high conductivity, and a low bulk resistance of 61 ± 7 Ω cm^–1^. Moreover, the recycled conductive filament produced reproducible electrodes that were electrochemically characterized, showing a heterogeneous electron (charge) transfer rate constant (*k* ^0^_obs_) of (2.75 ± 0.12) × 10^–3^ cm s^–1^, improving that of conductive virgin polypropylene electrodes (2.05 ± 0.05) × 10^–3^ cm s^–1^. These electrodes were utilized in two electroanalytical setups developed for applications in clinical settings. First, the simultaneous electrochemical detection of acetaminophen (ACE) and phenylephrine (PHE) was investigated by using an external counter and reference electrode configuration. These analytes are commonly coformulated in over-the-counter cold and flu medications, highlighting the importance of their concurrent quantification for pharmaceutical quality control and clinical analysis. Second, the sensing of uric acid (UA) using printed electrodes for the working, counter, and reference electrodes, achieving a limit of detection of 0.03 μM and achieving a recovery of 97.6% in urine, sensing of uric acid in urine is important as it is a biomarker for illnesses, for example, gout. This work highlights how waste PP from high use sectors can be upcycled to added-value products, with excellent performance, while contributing toward a circular economy electrochemistry.

## Graphical abstract

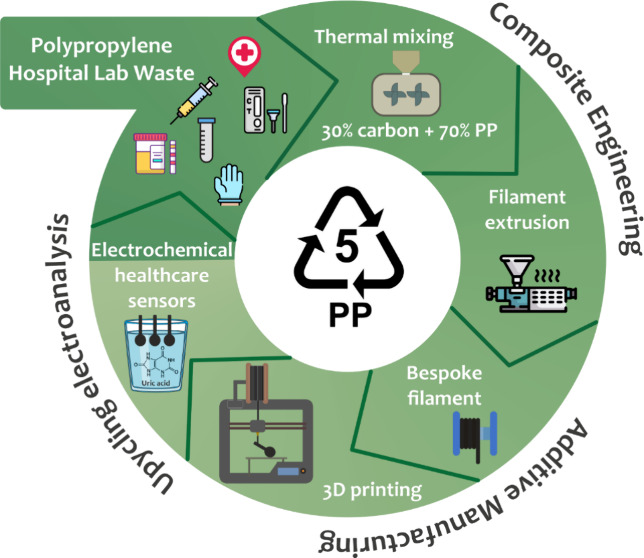

## 1 Introduction

Sustainable development is most often quoted to be “development that meets the needs of the present without compromising the ability of future generations to meet their own needs” and has become an increasingly important topic of discussion among governing bodies around the globe.^1^ The United Nations Sustainable Development Goals (SDGs) look to address this directly with 17 primary goals spanning a range of topics, such as Goal 3 “Good Health and Well-Being” or Goal 12 “Responsible Consumption and Production”.^2^ One key challenge tackled by Goal 12 is plastic waste, which is a growing chronic global problem.^3–5^ Within the United Kingdom, the National Health Service (NHS) has been urged by the Department of Health and Social Care (DHSC) to make a necessary shift away from wasteful practices.^6^ Following the Covid-19 pandemic, the NHS experienced a surge in plastic consumption, generating around 133,000 tons of plastic waste annually, in Great Britain.^6^ This trend was seen globally within healthcare during this time, exacerbating the need for innovative solutions.^7^

A significant amount of plastic waste is single-use plastics (SUPs), which have become an integral part of hospital operations due to their convenience, hygiene, and cost-effectiveness. Common uses of SUPs in hospital include gloves, syringes, sample pots, pipettes, and surgical trays.^6^ However, this heavy reliance on disposable plastics presents concerns as a significant amount of this waste is disposed of into landfill.^8^ Furthermore, the production of SUPs contributes toward carbon emissions and resource depletion, as they are made from virgin plastics, further straying from the environmental targets, such as net zero. The most used plastics are polyolefins, which include poly­(propylene) (PP), low-density poly­(ethylene) (LDPE), high-density poly­(ethylene) (HDPE), and poly­(ethylene terephthalate) (PET). HDPE, LDPE, and PET are formed through the polymerization of ethylene, which is produced through the steam cracking of petroleum and natural gases. Polypropylene is made through the chain-growth polymerization of propylene, which is a byproduct of the polymerization of ethylene. The propylene monomer is subjected to heat and pressure, in the presence of a catalyst, leading to bonding to form longer chains, resulting in polypropylene.^9,10^ These processes utilize sources that are not renewable, further reinforcing the need for a push away from unsustainable practices that exhaust limited resources.

As such, upcycling for plastics is crucial to address these issues of resource depletion and make strides toward reaching the SDGs, with the European Commission adopting this as a key strategy in 2018.^11^ Upcycling is a technique in which materials are recycled and reused to produce a new product, thus minimizing waste and maximizing resource efficiency.^12^ The importance of upcycling drastically increased during and following the pandemic with an increase in consumption of personal protective equipment, which is commonly made of plastics.^13^ In the literature, it can be seen that numerous techniques have been used to demonstrate how recycling can be done. For example, Ghabezi et al. demonstrated that material extrusion can be a pathway for plastic recycling, showcasing the transformation of HDPE domestic thermoplastic waste into components with desirable mechanical properties as well as optimizing the printing parameters for material extrusion.^14^ Following this, Sam-Daliri et al. showed how injection molding can be used to upcycle components originally fabricated via material extrusion, transforming them into functional composites while working within a circular economy framework.^15^ By using upcycling in hospitals, they can play a leading role in advancing environmental practices as well as ensuring that patient safety and care is not compromised.

Hospitals are major contributors to plastic waste, and a large amount of this is polypropylene. This is because polypropylene possesses ideal qualities such as excellent chemical stability,^16^ high-temperature resistance,^17^ lightweight,^18^ sterilizable, and durable, which are all key properties when working in medical procedures as it ensures safety and integrity. Due to these important features, one potential method to reuse plastic waste is additive manufacturing, which is the name given to the process by which an object is created via the addition of subsequential layers, opposed to traditional subtractive methods.^19^ Additive manufacturing, in particular Fused Filament Fabrication (FFF), is a highly versatile method and offers several advantages such as easy customization, reduced waste, fast prototyping, and on-site and on-demand production, all at low-cost.^20,21^

Researchers in electrochemistry have adopted FFF as a method to produce working electrodes^22^ and equipment^8,23,24^ through the use of commercially available conductive filament. These produced functional components; however, there were limitations around these filaments, such as slow electrochemical response, low sensitivity, and inadequate capabilities of sensing. Improvements in performance were sought through methods such as post-print surface activation;^25–28^ however, this did not lead to improvements significant enough to compete with conventional electrodes. Hence, the production of bespoke conductive filaments^29^ became a priority in the field. Researchers began by developing bespoke poly­(lactic acid) (PLA) filaments in conjunction with conductive fillers such as carbon black.^30–33^ This resulted in a filament that was significantly better than that of commercial filament. Further improvements were also made by incorporating additives such as carbon nanotubes,^34,35^ graphite,^36–41^ functional fillers,^42–44^ metallic nanoparticles,^45–48^ and Mxenes.^49^ Carbon black (CB) is one of the most used conductive fillers in additive manufacturing electrochemistry mainly due to CB having a smaller particle size, which allows for better dispersion and a more homogeneous distribution within the polymer matrix. The smaller particle size also contributes to a less disruptive extrusion process, as using other carbon morphologies such as carbon nanotubes or graphite powder can often lead to blockages or clogging of the nozzle. Moreover, in terms of cost-effectiveness, CB is significantly cheaper to other carbon morphologies, like carbon nanotubes, minimizing the cost of scaling-up production of conductive filaments. In a recent publication, Khan et al. provided with a more detailed comparison of different carbon morphologies to produce additive manufactured electrodes.^50^ Moreover, circular economy conceptualization has been also implemented in additive manufacturing electrochemistry where researchers have shifted toward using recycled PLA,^51,52^ which has shown comparable performance to virgin PLA. The use of alternative bio-based plasticizers^53–56^ for filament production has been also investigated while also adhering to sustainable development. However, whether recycled or not, conductive PLA faces issues due to the intrinsic features of this polymer that has resulted in limitations with providing reliable results following sterilization^57^ and poor chemical stability,^58^ as well as allowing a significant amount of ingress,^59^ essentially making the additively manufactured electrodes produced of single use.

Bespoke filaments have now advanced to where other polymers, such as polypropylene (PP),^60,61^ poly­(ethylene terephthalate glycol) (PETg),^57,62^ and thermoplastic polyurethane (TPU)^63,64^ are being adopted to provide desired characteristics and properties needed for the application of the electrodes. Initial work completed by Ramos et al.,^61^ demonstrated for the first time how polypropylene can be made into a conductive polymer and how it can open new avenues for researchers. In the study, it was seen that a polypropylene electrode can remain intact after being submerged in various aqueous and non-aqueous (organic) solvents for a period of 15 days. This finding establishes a significant advancement in additive manufacturing electrochemistry as previously, when working with PLA, organic solvents and non-aqueous solution were not possible to use. Furthermore, PP does not allow for high levels of ingress, unlike PLA, meaning that electrodes can be reliably used multiple times, leading to less waste created.

This paper presents the first reported fabrication of a conductive recycled polypropylene filament derived from hospital laboratory waste, marking a significant advancement in sustainable materials development. Through the application of additive manufacturing, the filament is used to produce electrodes tailored for two distinct healthcare-related sensing applications: (i) the simultaneous detection of acetaminophen and phenylephrine and (ii) the sensing of uric acid in urine using a fully integrated platform. This work not only demonstrates the feasibility of using recycled polypropylene as a viable alternative to virgin polypropylene but also contributes to the growing field of sustainable additive manufacturing for electrochemical sensing, offering a practical solution to the escalating issue of polypropylene waste in clinical environments.

## 2 Experimental Section

### 2.1 Chemicals

All chemicals used were of analytical grade and used as received without any further purification. All solutions were prepared with deionized water of resistivity not less than 18.2 MΩ cm from a Milli-Q Integral 3 system from Millipore UK. Hexaamineruthenium­(III) chloride (98%), potassium ferricyanide (99%), potassium ferrocyanide (98.5%), potassium chloride (99%), sodium hydroxide pellets (97%), calcium chloride (96%), sodium chloride, potassium phosphate monobasic (99%), ammonium chloride (99.5), urea (100.5%), acetaminophen (99%), and uric acid (99%) were purchased from Sigma (Gillingham, UK). Creatinine (98%), R(−)-phenylephrine hydrochloride (98%), was purchased from Fisher Scientific (Loughborough, UK). Carbon black was purchased from PI-KEM (Tamworth, UK), Virgin poly­(propylene) (PP, Sabic CX03–81 Natural 00900) was purchased from Hardie Polymers (Glasgow, UK), and recycled hospital lab polypropylene was supplied by RecycleLab (London, UK).

### 2.2 Bespoke Filament Production

Prior to any procedure for filament production, all PP pellets were dried in an oven at 80 °C for a minimum of 2.5 h to remove any residual water within the polymer. The bespoke conductive filament was prepared using 70 wt % PP and 30 wt % CB without the need of additional plasticizers. The compounds were mixed at 210 °C with Banbury rotors for 5 min by using a Thermo-Haake Polydrive dynameter fitted with a Thermo-Haake Rheomix 600 (Thermo-Haake, Germany). The resulting polymer composite was allowed to cool to room temperature before being granulated to create a finer granule size by using a Rapid Granulator 1528 (Rapid, Sweden). The granulated samples were collected and processed through the hopper of an EX2 extrusion line (Filabot, VA, USA). The molten polymer was extruded from a 1.75 mm die head and pulled along an Airpath cooling line (Filabot, VA, USA). Filament was then measured by using calipers to ensure that the filament had been extruded at a stable diameter within an acceptable tolerance range. The filaments were then ready to be used for additive manufacturing.

### 2.3 Additive Manufacturing of Electrodes

All computer designs and .3MF files in this article were produced using Fusion 360® (Autodesk®, CA, USA). These files were sliced and converted to .GCODE files and were taken to for printing by the open-source software PrusaSlicer (Prusa Research, Prague, Czech Republic). The additively manufactured electrodes were 3D-printed using fused filament fabrication (FFF) technology on a Prusa i3MK3S+ (Prusa Research, Prague, Czech Republic). All additively manufactured electrodes were printed onto Magigoo® glue using a 0.6 mm nozzle with a nozzle temperature of 220 °C and bed temperature of 100 °C, 100% rectilinear infill,^65^ 0.15 mm layer height, and print speed of 35 mm s^–1^. A 0.6 mm nozzle was chosen to be used to avoid any potential blockages while printing, which is more likely to occur when using a 0.4 mm nozzle. A schematic of the electrode preparation process can be found in the following referenced papers.^60,61^ Figure S1 shows a real image of an additively manufactured electrode used in this work.

### 2.4 Physicochemical Characterization

Scanning electron microscopy (SEM) micrographs were obtained using a Crossbeam 350 Focused Ion Beam-scanning electron microscope (FIB-SEM) (Carl Zeiss Ltd., Cambridge, UK) fitted with a field emission electron gun. Imaging was completed using a secondary electron ion (SESI) detector. Samples were mounted on the aluminum SEM pin stubs (12 mm diameter, Agar Scientific, Essex, UK) using adhesive carbon tabs (12 mm diameter, Agar Scientific, Essex, UK) and coated with a 3 nm layer of Au/Pd metal using a Leica EM ACE200 coating system before imaging.

Raman spectroscopy was performed on a Renishaw PLC in Via Raman Microscope controlled by the WiRE 2 software at a laser wavelength of 514 nm.

X-ray photoelectron spectroscopy (XPS) data were acquired using an AXIS Supra (Kratos, UK), equipped with a monochromated Al X-ray source (1486.6 eV) operating at 225 W and a hemispherical sector analyzer. It was operated in fixed transmission mode with a pass energy of 160 eV for survey scans and 20 eV for region scans with the collimator operating in slot mode for an analysis area of approximately 700 × 300 μm; the FWHM of the Ag 3d5/2 peak using a pass energy of 20 eV was 0.613 eV. Before analysis, each sample was ultrasonicated for 15 min in propan-2-ol and then dried for 2.5 h at 65 °C as shown in our unpublished data to remove excess contamination and minimize the risk of misleading data. The binding energy scale was calibrated by setting the graphitic sp^2^ C 1s peak to 284.5 eV;^66^ this calibration was acknowledged to be flawed but was nonetheless used in the absence of reasonable alternatives, and because only limited information was to be inferred from absolute peak positions.

Contact angles were assessed using a bespoke additive manufactured printed measurement setup consisting of an additive manufactured stage, a Hamilton syringe, and a generic USB digital microscope connected to a computer. This equipment allowed consistent delivery of droplets to part surfaces, which could be recorded as video and/or photographs using the microscope. Where possible, contact angles were analyzed using ImageJ and the DropSnake plug-in, with reported values being the average and standard deviation of three repeat measurements.

### 2.5 Electrochemical Experiments

All electrochemical measurements were performed on an Autolab 100N potentiostat controlled by NOVA 2.1.6 (Utrecht, the Netherlands). The electrochemical characterization of the bespoke filament and comparison to the benchmarks were performed using a lollipop design (Ø 5 mm disc with 8 mm connection length^67^ and 2 × 1 mm thickness) electrodes alongside an external commercial Ag|AgCl (3 M KCl) reference electrode with a nichrome wire counter electrode.

Activation of the additively manufactured electrodes was performed before the electrochemical experiments. This was achieved electrochemically in NaOH.^27^ The additively manufactured electrodes were used as a working electrode alongside a nichrome wire coil as a counter electrode and Ag|AgCl (3 M KCl) as a reference electrode and placed into a 0.5 M NaOH solution. Chronoamperometry was used to activate the additively manufactured electrodes by applying a set voltage of +1.4 V for 200 s, followed by applying −1.0 V for 200 s. The additively manufactured electrodes were then thoroughly rinsed with deionized water and dried under compressed air before further use.

The conditions for capacitance experiments were as follows: start potential = +0.15 V; upper potential = +0.35 V; step potential = 0.00244 V; and scan rate = 0.01–0.3 V/s. Conditions for electrochemical impedance spectroscopy were frequency 0.1 Hz–100 kHz and amplitude 0.01 V, and then repeated five times per electrode. Electroanalytical applications were performed using differential pulse voltammetry (DPV) with the following parameters: acetaminophen and phenylephrine start potential = +0.0 V, stop potential = +1.2 V, step potential = 0.008 V, modulation amplitude = 0.08 V, modulation time = 0.05 s, and interval time = 0.5 s and uric acid—start potential = +0.0 V, stop potential = +1.0 V, step potential = 0.008 V, modulation amplitude = 0.08 V, modulation time = 0.05 s, and interval time = 0.5 s. Spiked synthetic urine samples were analyzed through the external calibration method, where the synthetic urine was spiked with 30 μM of uric acid and diluted in 0.01 M PBS (pH = 7.2, 1:20).

## 3 Results and Discussion

### 3.1 Physicochemical Characterization

Thermal mixing techniques were used to produce the filaments as outlined in Section 2. This approach was used as opposed to solvent mixing as it allows for less waste to be produced and a more homogeneous mix as the shear force produced during the mixing allows for a more even distribution of the carbon black throughout the polymer. The filaments were made using polypropylene (70 wt %) as the base polymer and carbon black (30 wt %) as the conductive filler, with the resulting bespoke filaments inheriting excellent levels of flexibility as they would allow for significant manipulation without breaking. The percolation threshold for CB/PP has been previously demonstrated by Ramos et al.; however, in this work, a 30% carbon black loading was used as when higher loading, such as 32.5%, was prepared, the conductive filament became noticeably more brittle and made the additive manufacturing of the electrodes difficult.^61^ Once the percolation threshold was met and enough carbon black had been introduced into the polymer mix, the formation of a conductive network occurred. The carbon black particles formed a continuous interconnected network through the polymer matrix, which created conductive pathways that allowed electrons to move through the otherwise insulating polypropylene. Figure S2A,B shows images of both the virgin and hospital lab waste (hospital waste) conductive filaments and their flexibility. Then, the resistance of the filaments was measured across 10 cm segments in five different sections of the filaments with virgin polypropylene displaying a bulk resistance of 48 ± 2 Ω cm^–1^, compared to hospital waste filament, which showed a resistance of 61 ± 7 Ω cm^–1^. The filaments were then used to additive manufactured electrodes that were next characterized by SEM, Raman, and XPS. Following the production of the electrodes, they were activated, as described in Section 2.5, which is a common method used to enhance the electrochemical response. This is due to the procedure stripping away polymer, which had migrated to the surface during the extrusion, which in turn leads to a higher surface area of the electrodes. However, in the case of PP, activation was a necessary step that needed to be taken as electrodes were printed onto an adhesive that was successfully removed upon this procedure and ensured optimal electrode performance.

Contact angle experiments were completed on the resulting virgin and hospital waste electrodes to understand their interactions with water. Figure S3A,B shows images of how the water droplet interacted with the respective electrodes. It was calculated that the virgin electrode had a contact angle of 99 ± 3° compared to the hospital waste electrode, which had an angle of 94 ± 1°. These results indicated that both materials had hydrophobic tendencies, with the virgin material showing slightly more hydrophobicity. This reinforced the notion that the ingress of the solution was minimized when using polypropylene, leading to an electrode that can endure further use.

Physicochemical analysis of the additively manufactured electrodes was first done by using SEM, which is shown in Figure 1A,B. These images show similarities between the two polymers and how the carbon filler is dispersed throughout the electrodes; however, it is noticeable that an increase in surface perforations is seen in the hospital waste electrode. This is expected due to the lower quality of material found within recycled polymers. The surface chemistry of the conductive filaments was analyzed by XPS, specifically looking at the C 1s regions, as seen in Figure 1C for the virgin electrode and Figure 1D for the hospital waste electrode showing a clear peak at 284.5 eV, which is associated with the X-ray photoelectron emission by graphitic (sp^2^) carbon.^68,69^ The atomic concentration for the graphitic carbon peak of the virgin electrodes, 63.57%, was found to be higher than that of the hospital waste electrodes, 56.42%. Graphitic carbon allowed for delocalized electrons to move freely; therefore, an increase in concentration of these carbons allowed for better electronic conductivity. Raman analysis was next performed on the virgin and hospital waste electrodes. Figure 1E shows the comparison of the graphitic like peaks for both polymers, with intense peaks at 1338, 1572, and 2680 cm^–1^ assigned to the D-, G-, and 2D-bands, respectively.^70,71^ The *I*_*D*_/*I*_*G*_ ratio for these peaks was calculated to be 1.04 for virgin and 1.05 for hospital waste. Ratios closer to 0 indicated a very ordered structure, whereas a ratio closer to 1 indicated towards a less ordered structure, which was what both these polymers exhibited. This response was expected as carbon black has an amorphous structure unlike graphite or graphene.

**Figure 1:**
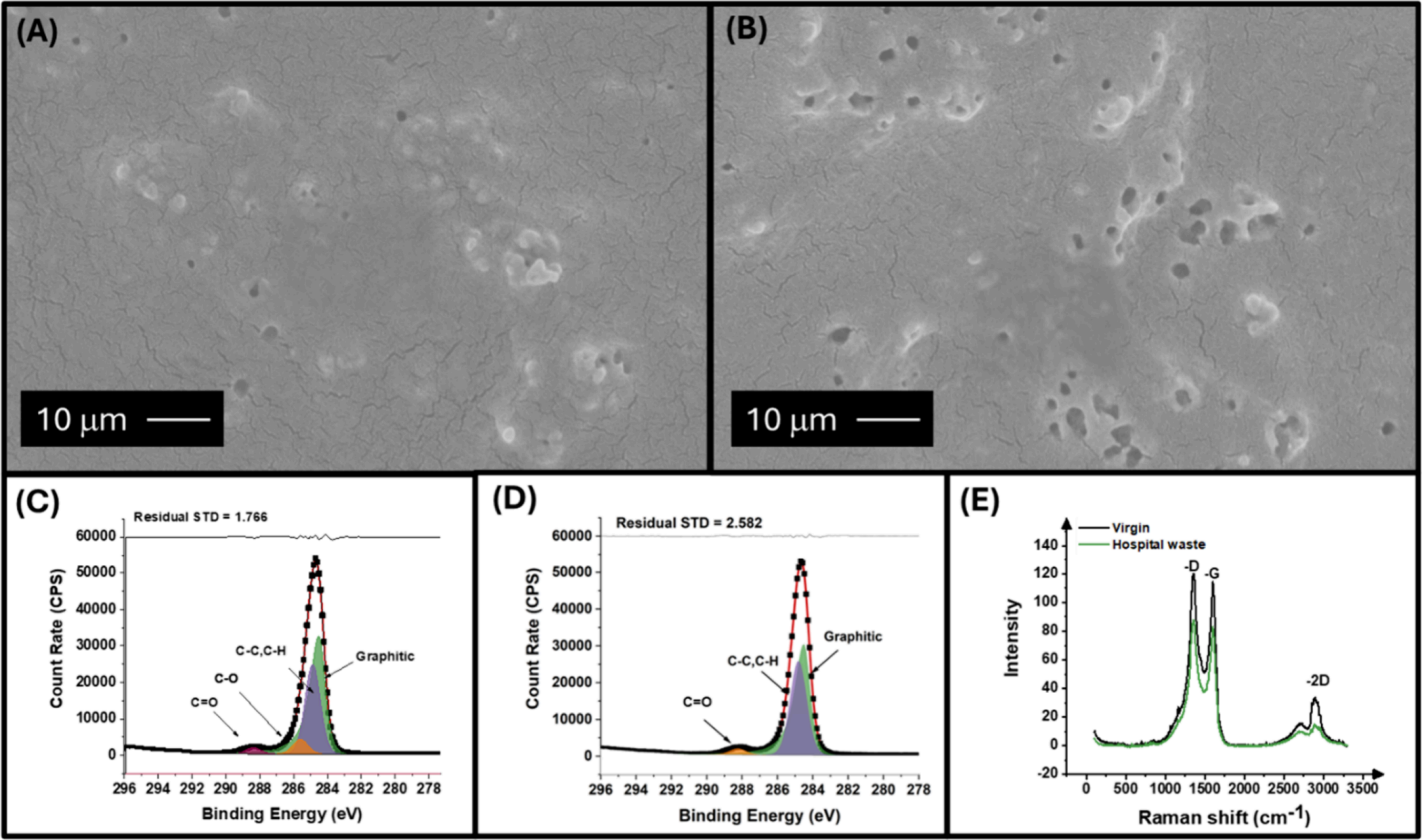
SEM surface images for the (A) virgin electrode and (B) hospital waste electrode. XPS C 1s region for the (C) virgin and (D) hospital waste electrode. (E) Raman spectroscopy data for virgin (black) and hospital waste (green).

### 3.2 Electrochemical Characterization

Following physicochemical characterization of the additively manufactured electrodes, it was necessary to electrochemically characterize the electrodes. Firstly, to understand if the recycling of polypropylene causes a variation in the materials that would impact the electrochemistry of the electrodes, cyclic voltammetry (CV) was used in 0.1 M KCl to determine and compare the potential windows for both materials. Figure 2A shows that both electrode types share the similar potential window from −1.8 to +1.8 V, suggesting that they can be both applied within the same potential range without compromising the analysis. Following this, determination of the double-layer capacitance (*C*_dl_) of the electrodes produced using both virgin and hospital waste polypropylene allowed for comparing their electrochemical performance. This calculation was performed upon analyzing cyclic voltammogram obtained after varying scan rates within the non-Faradaic region (Figure S4A,B). It was calculated that the virgin electrode had a capacitance of 0.64 ± 0.18 μF compared to the hospital waste electrodes, which had a capacitance of 0.96 ± 0.05 μF. Although similar values have been obtained for the virgin and hospital waste electrodes suggesting that using recycled polypropylene shows no significant effect on the electrode’s capacitive abilities, the slightly higher value obtained for hospital waste electrodes could involve an increased electroactive area and, therefore, an improved electrochemical performance when used in electroanalytical applications

**Figure 2:**
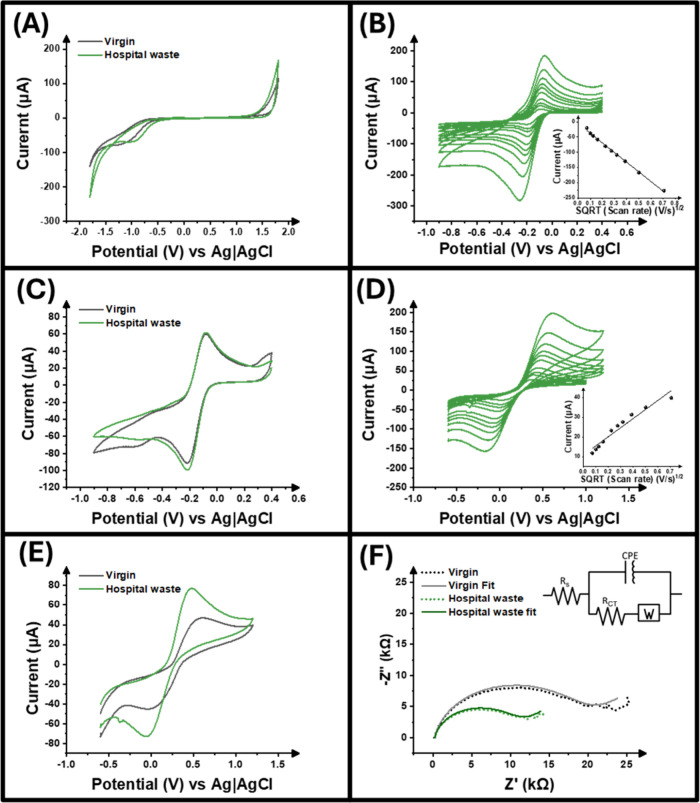
(A) Cyclic voltammogram (50 mV s^–1^) showing the potential window for the virgin (black) and hospital waste (green) electrodes in 0.1 M KCl. (B) Scan rate study (0.005–0.50 V s^–1^) in [Ru­(NH_3_)_6_]^3+^ (1 mM in 0.1 M KCl) performed on hospital waste electrode. (C) Cyclic voltammogram (50 mV s^–1^) comparing virgin and hospital waste electrodes in [Ru­(NH_3_)_6_]^3+^ (1 mM in 0.1 M KCl). (D) Scan rate study (0.005–0.500 V s^–1^) in [Fe­(CN)_6_]^3-^ (1 mM in 0.1 M KCl) performed on hospital waste electrode. (E) Cyclic voltammogram (50 mV s^–1^) comparing virgin and hospital waste electrodes in [Fe­(CN)_6_]^3–^ (1 mM in 0.1 M KCl). (F) Comparison of EIS Nyquist plots for virgin and hospital waste electrodes in 1 mM [Fe­(CN)_6_]^3–^ 0.1 M KCl.

To continue the electrochemical characterization, electrodes were tested against the common outer sphere, [Ru­(NH_3_)_6_]^3+^, and inner sphere, [Fe­(CN)_6_]^3–^ redox probes. Through these experiments, there is better understanding of the differences in the kinetics of the electrode upon employing different base polymers, as they allow for the calculation of the heterogeneous electron (charge) transfer rate constant (*k* ^0^_obs_) and electrochemical area (*A*_*e*_). The *k* ^0^_obs_ were calculated as an average of 3 sets of 10 different scan rate experiments recorded from 5 to 500 mV s^–1^, where each set used a new additively manufactured electrode. To obtain the results, the Nicholson method was used for quasi-reversible electrochemical reactions through eq 1:^72^

$$\Psi ={k^{0}}_{\mathrm{obs}}{\left(\pi DnvF/\mathrm{RT}\right)}^{-1/2}$$ 1

where Ψ is a kinetic parameter, *D* is the diffusion coefficient ([Ru­(NH_3_)_6_]^3+^ of 9.10 × 10^–6^ cm^2^ s^–1^ ^73^, [Fe­(CN)_6_]^3–^ of 7.26 × 10^–6^ cm^2^ s^–1^ ^74^), *n* is the number of electrons that are taking part in the electrochemical reaction (1), *F* is the Faraday constant (96,485 C mol^–1^), *v* is the scan rate (5-500 mV s^–1^), *R* is the universal gas constant (8.314 J mol^–1^ K^–1^), and *T* is the temperature in Kelvin (298 K). In order to calculate *k* ^0^_obs_, the determination of the peak-to-peak separation (Δ*E_p_*) must be completed to then deduce Ψ, where Δ*E_p_* is obtained at various voltammetric scan rates. The *k* ^0^_obs_ value can then be obtained via the gradient when plotting Ψ against [πDnvF/RT]^−1/2^.

Figure 2B shows the response of the hospital waste electrodes when used in [Ru­(NH_3_)_6_]^3+^ across scan rates of 0.005–0.5 V s^–1^; it can be said that the redox reaction is diffusion-controlled, which is reinforced by the Randles-Ševčík inset plot in Figure 2B. The data obtained from this experiment is summarized in Table 1. As stated previously, through the use of the Nicholson equation, it was calculated that the virgin electrodes had a *k* ^0^_obs_ of (2.05 ± 0.05) × 10^–3^ cm s^–1^ compared to that of the hospital waste electrodes of (2.75 ± 0. 12) × 10^–3^ cm s^–1^. The higher *k* ^0^_obs_ for the hospital waste electrodes can be attributed to the more porous structure of the material, which is due to the lower grade of polymer used as a result of being a recycled material. Due to being a lower grade polymer, it is more susceptible to degradation under heat, which leads to pores within the material being formed, which allow for better access to the carbon filler. As well as this, by having pores in the material, it results in the higher surface area. Table 1 confirms this also, as it shows that the hospital waste electrodes have a slight increase (0.08 cm^2^) in the electrochemical surface area compared to the virgin electrodes, which is also in agreement with the double-layer capacitances previously calculated. A visual comparison of both the virgin and hospital waste electrodes’ response at 0.05 V s^–1^ can be seen in Figure 2C, showing almost identical voltammograms, with the hospital waste electrode having a minimally increased reduction peak, which is to be expected as the data derived from the scan rate experiment shows the hospital waste electrode giving an increased electrochemical response.

**Table 1:** Compilation of Data Derived from Scan Rates Studies in [Ru­(NH_3_)_6_]^3+^ and [Fe­(CN)_6_]^3–^, Highlighting the Heterogenous Electron Transfer Rate (*k* ^*0*^_obs_) and Electrochemical Surface Area (*A_e_*)

Electrode	*k* ^0^_ob*s*_ (cm s^–1^) [Ru(NH_3_)_6_]^3+^	*A*_*e*_ (cm^2^) [Ru(NH_3_)_6_]^3+^	*I_p_* Ox (μA [Fe(CN)_6_]^3–^	*A*_*e*_ (cm^2^) [Fe(CN)_6_]^3–^
Virgin	(2.05 ± 0.05) × 10^–3^	0.35 ± 0.01	31.0 ± 7.21	0.17 ± 0.04
Hospital waste	(2.75 ± 0.12) × 10^–3^	0.43 ± 0.02	60.9 ± 2.32	0.29 ± 0.13

Additionally, scan rate studies in [Fe­(CN)_6_]^3–^ were performed, with Figure 2D showing the voltammograms from this experiment for the hospital waste electrodes. Data derived from this experiment are included in Table 1. When comparing values calculated for peak current for the virgin and hospital waste electrodes using the inner redox probe, it can be seen that the virgin electrodes perform worse with a peak current of 31.0 ± 7.21 μA, compared to that of the hospital waste electrodes, which had 60.9 ± 2.32 μA. This is also demonstrated in Figure 2E, which visualizes the hugely increased current response. This response can be supported by calculating the electrochemical surface area, as an increased area would lead to an increased current response. The hospital waste electrode was calculated to have the greater area of 0.29 ± 0.03 cm^2^ compared to the virgin, which had 0.17 ± 0.04 cm^2^. This data further reinforces the notion that the recycled polypropylene electrodes have an increased area due to the lower grade of polymer supporting more solution accessing carbon black active sites.

To complete the electrochemical characterization, electrochemical impedance spectroscopy (EIS) was performed on both electrodes in [Fe­(CN)_6_]^3–^ (1 mM in 0.1 M KCl), as represented in Figure 2F. Through the use of EIS, there is an understanding of the differences in solution resistance (*R_S_*) and charge transfer resistance (*R*_CT_) of the two electrode types. It was seen that the *R_S_* increased when comparing the virgin electrode to the hospital waste electrode, from 191.3 ± 9.4 to 424.2 ± 83.3 Ω, which is in accordance with the resistances seen for the filaments previously where the virgin material showed a lower value. The *R*_CT_ for the virgin electrode was 15.7 ± 2.8 kΩ, and for the hospital waste electrode, this decreased to 10.7 ± 2.5 kΩ. This decrease can be attributed to the lower quality of the recycled polymer, creating easier access to the conductive filler and therefore aiding charge transfer.

Due to the interest of this work surrounding the subject of recycling, it is important to showcase that the electrodes are feasible for multiple uses, to minimize waste produced. For this reason, the electrochemical stability of the polymers in the solution was investigated through use of cyclic voltammetry (in [Ru­(NH_3_)_6_]^3+^ (1 mM in 0.1 M KCl)) to provide an insight on the longevity of electrodes when used in an electrochemical environment. Electrodes were tested across 100 scans at 50 mV s^–1^. Data collected from this experiment is represented in Figure S5. It is evident that both materials showed similar responses throughout the experiment, with a plateau occurring across the initial 50 scans when looking at peak current, before a significant drop off in the peak current in the following 50 scans. Across the 100 scans, the virgin electrode had a higher percentage decrease of 52.4% compared to the hospital waste electrode, which had 45.7% drop off. This data provides valuable insight into the longevity of polypropylene electrodes, both virgin and recycled, and how they are viable as potential multi-use items. The ability to reuse electrodes is a characteristic that PLA does not possess due to the large amount of ingress the polymer faces,^59^ ultimately making it a single-use item, as opposed to polypropylene.

### 3.3 Study of Electrochemical Performance upon Sterilization

Sterilization of equipment used in hospital settings or clinical applications is a crucial step as it prevents cross contamination, ensures patient safety, and minimizes the risk of infections. To demonstrate if the additively manufactured electrodes developed in this work could have a potential application in medical scenarios while maintaining their electrochemical performance, it is important to investigate them upon applying common sterilization methods, as previously described in the literature for PETg-based sensors.^57^ Briefly, additively manufactured electrodes produced from both virgin and hospital waste PP filaments were placed within a benchtop Anycubic Wash and Cure Plus station and irradiated with UV light for 4 h. Then, sterilized electrodes were electrochemically tested against the same inner sphere probe used previously so that a comparison with the non-sterilized electrodes can be established.

Figure 3 A,B illustrates the comparison of the cyclic voltammograms obtained for the virgin and hospital waste electrodes upon UV sterilization. When comparing the before and after images for the virgin electrodes, it is apparent that no significant changes occur. This is confirmed by data included in Table 2, which shows that there is a minimal increase of 11.8 μA in the peak current of virgin electrodes following the process of sterilization. Similarly, no statistically significant changes are observed in the electrochemical area of the electrodes produced from virgin polypropylene filament upon sterilization. The same behavior can also be seen for the hospital waste electrodes, with peak current increasing only by 9.5 μA and electrochemical surface area by 0.01 cm^2^, as described in Table 2. This data confirms that both materials regardless of the quality of the polypropylene-based polymer remain stable throughout the process of sterilization and ensures that the electrochemical devices can be used in a medical environment as their sterilization does not affect their electrochemical performance.

**Figure 3:**
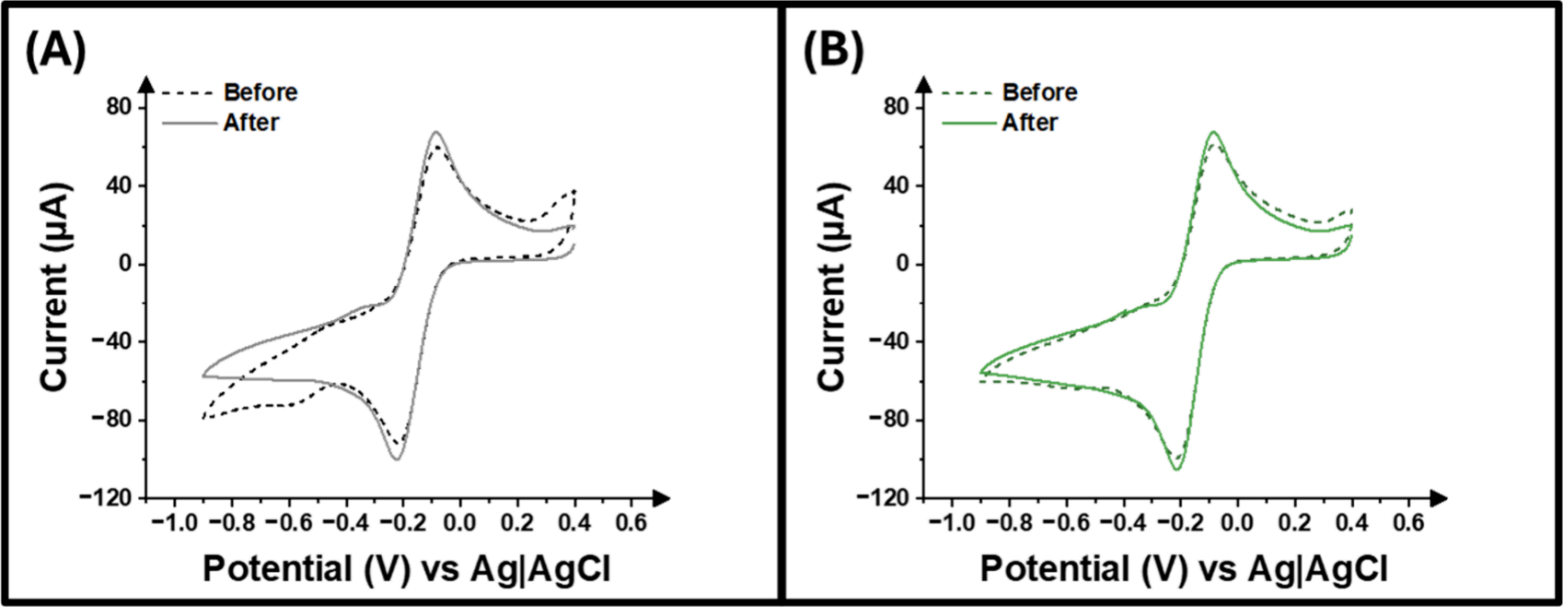
Cyclic voltammogram (50 mV s^–1^) comparing (A) virgin and (B) hospital waste electrodes in [Ru­(NH_3_)_6_]^3+^ (1 mM in 0.1 M KCl) before and after sterilization.

**Table 2:** Compilation of Data Derived from Scan Rate Studies in [Ru­(NH_3_)_6_]^3+^ Before and After Sterilization of Virgin and Hospital Waste Electrodes, Highlighting the Peak Current (*I_p_*) and Electrochemical Surface Area (*A*_*e*_)

	Before sterilization		After sterilization	
Eelectrode	*I_p_* (μA)	*A*_*e*_ (cm^2^)	*I_p_* (μA)	*A*_*e*_ (cm^2^)
Virgin	–66.5 ± 1.19	0.35 ± 0.01	–78.3 ± 5.88	0.36 ± 0.02
Hospital waste	–79.8 ± 5.41	0.43 ± 0.02	–89.3 ± 10.0	0.42 ± 0.03

### 3.4 Electroanalytical Applications

#### 3.4.1 Simultaneous Detection of Acetaminophen and Phenylephrine

Following the electrochemical characterization of the additively manufactured electrodes studied in this work, they were first applied to the simultaneous sensing of acetaminophen (ACE) and phenylephrine (PHE), two medically relevant compounds known for their synergistic effects in treating cold and flu symptoms and commonly found in over-the-counter formulations.^75,76^ The proposed electrochemical mechanisms for these analytes can be found in mechanisms S1 and S2. Furthermore, additively manufactured sensors that can provide simultaneous sensing opens access to low-cost, rapid options for point-of-care diagnostics of drug levels in biological fluids, leading to detection of misuse and improving patient safety.

Differential pulse voltammetry (DPV) was the chosen method for the simultaneous sensing of ACE and PHE, across a concentration range of 5–40 μM, as DPV provides an enhanced sensitivity and sharper, more well-defined peaks when compared to CV as the differential nature reduces background current and noise, making it a more favorable option for electrochemical analysis.^77,78^ Figure 4A,B shows the resulting DPV voltammograms recorded using the virgin and hospital waste electrodes with Figure 4C,D providing the calibration plots derived from the voltammograms registered at different concentration levels of ACE and PHE. Note that each calibration point was analyzed in triplicate. Both materials showed linear responses, with the virgin electrodes producing the equations *I*/μA = 0.047­[ACE] + 0.065 and *I*/μA = 0.071­[PHE] + 0.419. The hospital waste electrode produced the equations I/μA = 0.082­[ACE] + 0.0139 and I/μA = 0.111­[PHE] + 0.319. From the resulting calibration plots, the limit of detection (LOD) and limit of quantification (LOQ) were next estimated to evaluate the lowest concentration of the analytes that can be reliably quantified using the developed additively manufactured electrodes. LOD was calculated as three times the standard deviation of the blank divided by the slope of the calibration plot, while LOQ was calculated as 10 times the standard deviation of the blank divided by the slope of the calibration plot. The virgin electrode achieved an LOD and LOQ for PHE of 0.09 and 0.31 μM, respectively, compared to hospital waste, which achieved an LOD of 0.04 μM and an LOQ of 0.01 μM. On the other hand, for ACE, an LOD of 0.15 and 0.04 μM for virgin and hospital waste electrodes was observed, respectively, while the LOQ was calculated to be 0.52 and 0.14 μM for the virgin and hospital waste electrodes, respectively. This clearly demonstrates the improvement in sensitivity of the hospital waste electrodes for the simultaneous detection of ACE and PHE compared to that of virgin electrodes, which is also in agreement with the enhanced electrochemical performance of those electrodes investigated throughout this work. Finally, the sensitivity of the electrodes was also derived from the calibration plot, where both electrode types achieved excellent sensitivities toward ACE; the sensitivities were 0.04 and 0.08 μA μM^–1^, for the virgin and hospital waste electrodes, respectively, and for PHE, the sensitivity was calculated to be 0.07 μA μM^–1^ for the virgin PP and 0.11 μA μM^–1^ for the hospital waste PP. Comparisons to other sensors seen in literature for these analytes can be found in Tables S1 and S2. Through this comparison, it can be seen that the recycled hospital waste polypropylene electrodes showed better limits of detection towards both analytes, highlighting the significance of the results obtained.

**Figure 4:**
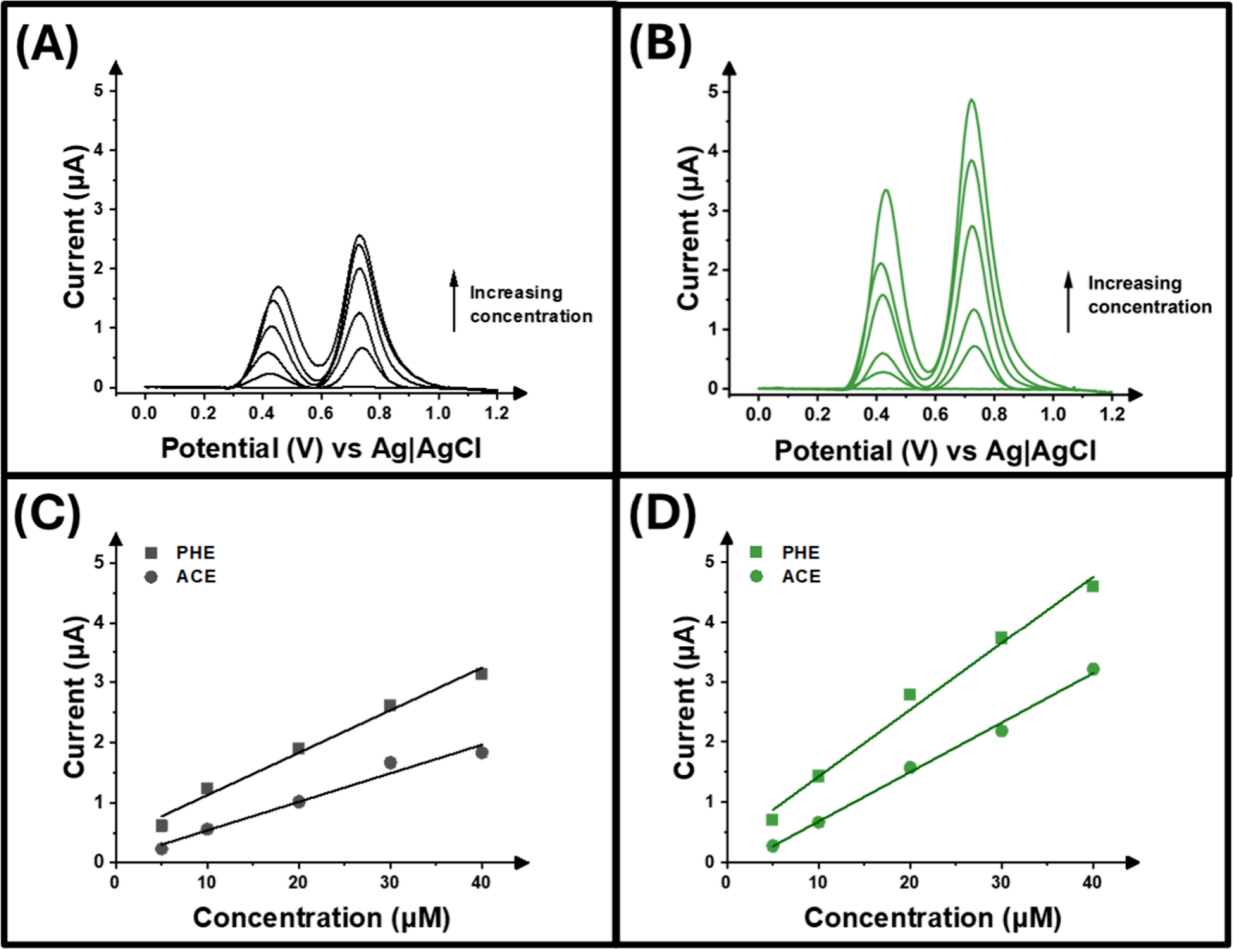
Differential pulse voltammogram for (A) virgin and (B) hospital waste electrodes for the simultaneous detection of phenylephrine and acetaminophen in a concentration range of 5–40 μM. Calibration plot for peaks obtained for phenylephrine (square) and acetaminophen (circle) using (C) virgin and (D) hospital waste.

#### 3.4.2 Detection of Uric Acid Using a Full Printed Setup

To move forward, the next step of transforming this technology into a feasible platform with real potential to be implemented in healthcare environments,^79^ we look to perform electroanalytical applications using a new setup where working, counter, and reference electrodes are additively manufactured using the conductive PP materials developed in this work. This setup was applied to the detection of uric acid (UA) in synthetic urine, highlighting its potential application for rapid, point-of-care diagnosis of urinary tract infections (UTIs). The electrochemical mechanism for this analyte can be found in Mechanism S3. Electrochemical sensing of UA in urine is particularly valuable due to its non-invasive, cost-effective, and rapid monitoring of health conditions. Increased levels of uric acid are associated with various medical problems, such as gout, kidney stones, and renal dysfunction, making UA a valuable biomarker for clinical diagnosis.^80^

To begin, a traditional set up, comprising additively manufactured working electrode, nichrome wire counter electrode, and Ag/AgCl reference electrode, was compared to the additively manufactured set up, involving additively manufactured working electrode (WE), counter electrode (CE), and reference electrode (RE). Figure S6 shows an expected peak potential shift for UA when it is analyzed using a fully additively manufactured setup, as demonstrated previously for other applications.^79^ Besides the observed shifting of the peak from +0.33 to +0.24 V, the application for UA determination was not affected, suggesting that the additively manufactured setup can replicate that of the traditional set up and that the redox environment at the surface is comparable. Both polymers were then used within the additively manufactured set up, where additively manufactured electrodes were used as the WE, CE, and RE, in a concentration range of 5–60 μM UA in 0.01 M PBS. Figure 5A,B shows that the peak currents increased with added concentration of the analyte and provided linear responses; the virgin electrodes produced a linear plot with the equation I/μA = 0.06­[UA] + 0.154, and the hospital waste electrodes produced a linear plot with the equation I/μA = 0.08­[UA] – 0.004. From the calibration plots, the limit of detection (LOD) and limit of quantification (LOQ) were calculated and found to be 0.04 μM and 0.14 μM for the virgin polypropylene, compared to the hospital waste polypropylene, which resulted in 0.03 μM and 0.12 μM, respectively. As well as this, the sensitivity of the electrodes was of interest; the virgin electrode achieved a sensitivity of 0.06 μA μM^–1^ and the hospital waste electrode achieved 0.08 μA μM^–1^. The high sensitivity of the electrodes showed that they can be applicable toward precise analysis while providing accurate responses. The recycled HW electrodes demonstrated that when applied towards uric acid, it showed a better limit of detection than that what was seen in literature. These comparisons of how the electrodes relate to sensors seen in the literature can be found in Table S3. Both types of electrodes provided data that has high linearity and repeatability when under lab conditions; however, it is important for this to be translated to conditions, which can be applied more towards real-world scenarios.

**Figure 5:**
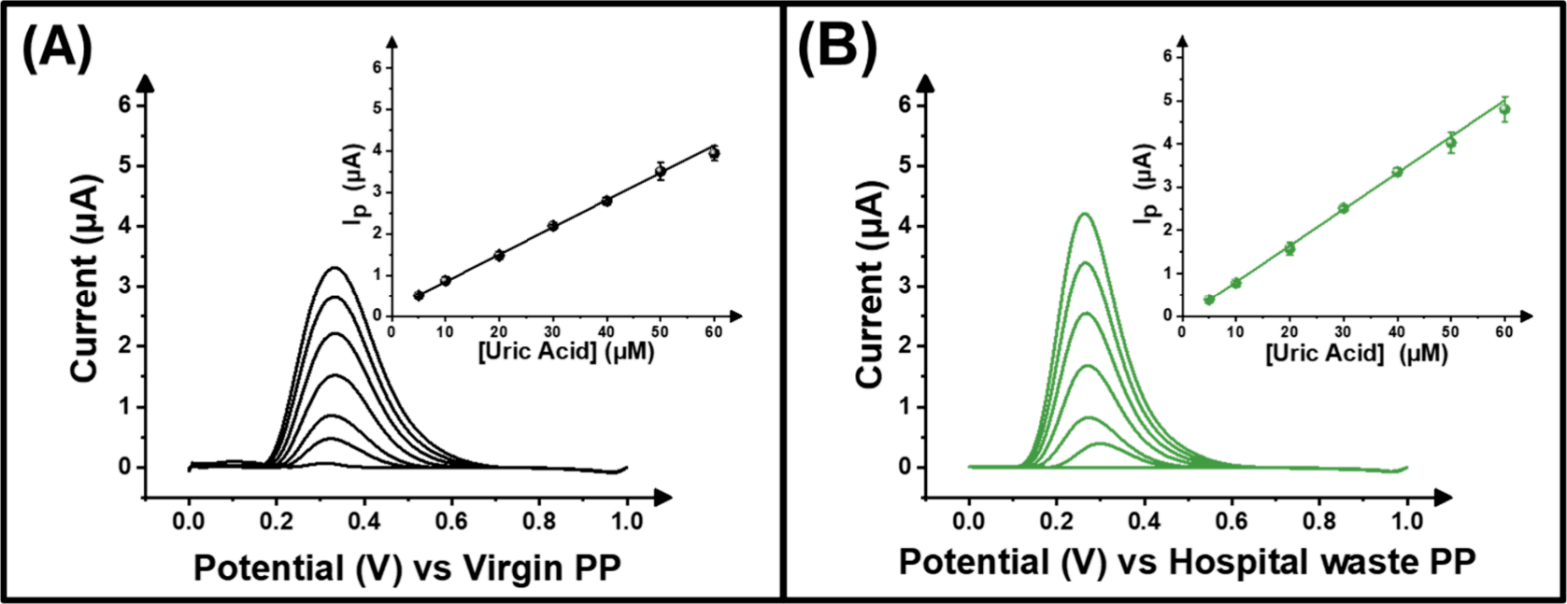
Differential pulse voltammogram for (A) virgin polypropylene and (B) hospital waste electrodes for the detection of uric acid in a concentration range of 5–60 μM in 0.01 M PBS. (Inset = calibration plot).

#### 3.4.3 Real Application in Synthetic Urine

To conclude, the additively manufactured set up was applied toward a real-life sample through the use of synthetic urine, which had been spiked with 30 μM of uric acid as shown in Figure S7A and B. The standard addition method was used to calculate a recovery value for each electrode type; it was determined that the virgin electrodes had a recovery of 103.9%, whereas the hospital waste electrode recovered a value of 97.6%. Both electrodes showed extremely encouraging results, as well as providing similar recovery rates, showing that recycled polypropylene was a viable alternative within electrochemistry and that using an all-in-one printed cell was an option that can be explored when using polypropylene electrodes.

## 4 Conclusions

In this work, we report for the first-time, use of recycled polypropylene for conductive additive manufacturing filaments. Hospital lab waste polypropylene was upcycled into additively manufactured electrochemical platforms by mixing the polymer with carbon black in a 70:30 wt % ratio, which was directly compared to a virgin polypropylene filament prepared in the same way. Both conductive filaments were used to 3D print electrodes, which were then initially physicochemically characterized using SEM, Raman, and XPS. Key results obtained in this work are listed as follows:

- Electrodes were electrochemically characterized, resulting in a *k* ^0^_obs_ of (2.05 ± 0.05) × 10^–3^ cm s^–1^ for the virgin electrodes and a *k* ^0^_obs_ (2.75 ± 0.12) × 10^–3^ cm s^–1^ for the hospital waste electrodes.
- Electrochemical characterization was completed through use of EIS, which gave clarity on charge transfer capabilities for both electrode types.
- It was shown that when both virgin and hospital waste electrodes were subjected to 100 scans of cyclic voltammetry; they remained stable up to 50 scans before seeing a drop off in performance, providing an understanding that these electrodes can be reusable.
- Stability testing performed by subjecting electrodes to UV treatment for 4 h revealed that polypropylene electrodes could be successfully sterilized without compromising the efficiency of the electrodes, as proven by the lack of significant change in peak current for both virgin electrodes (change of 11.8 μA) and hospital waste electrodes (change of 9.5 μA).

The materials were then applied to two distinct applications: (i) simultaneous detection of acetaminophen and phenylephrine and (ii) sensing of uric acid in synthetic urine using a fully additively manufactured setup:

- For the first application, LODs for the virgin material were obtained to be 0.15 and 0.09 μM for acetaminophen and phenylephrine, whereas the hospital waste electrode achieved LODs of 0.05 and 0.01 μM, respectively.
- For the second application, using the additively manufactured electrodes, recovery values of 103.9 and 97.6% were achieved for the virgin and hospital waste electrodes, respectively.

This work provides valuable insight on how hospitals can utilize upcycling to solve real-world problems such as sustainability and financial issues by opting toward using recycled materials in conjunction with additive manufacturing to produce cheap and effective electrodes for sensing applications, while still maintaining a high level of patient care and safety.

## Supplementary Material



## Notes

Images of the additive manufacture electrodes and filament; contact angle experiments; cyclic voltammograms obtained after varying scan rates within non-Faradaic region; analysis of cyclic voltammograms comparing virgin and hospital waste electrode across 100 scans; differential pulse voltammograms for a virgin electrode recorded in uric acid; differential pulse voltammograms for electrode for the recovery of uric acid in synthetic urine; mechanisms for acetaminophen, phenylephrine, and uric acid; table of comparison for the sensing of acetaminophen, phenylephrine, and uric acid (PDF)
