# Supplementary material for: Upcycling Hospital Lab Polypropylene Waste into a Fully Integrated Additive Manufacturing Electroanalytical Sensing Platforms
Source: ACS Sustain Resour Manag. 2025 Nov 3;2(11):2302–13. doi: 10.1021/acssusresmgt.5c00393 (PMC12676421; doi:10.1021/acssusresmgt.5c00393)
Supplement: Supplementary file 1 [file rm5c00393_si_001.pdf]

Supplementary Information for:

Upcycling Hospital Lab Polypropylene Waste into a Fully Integrated Additive Manufacturing Electroanalytical Sensing Platforms

Muhzamil A. Khan,[1] Elena Bernalte,[1] Danielle Stephens,[2] Robert D. Crapnell,[1*] and Craig E. Banks[1*]

[1] *Faculty of Science and Engineering, Manchester Metropolitan University, Dalton Building, Chester Street, M1 5GD, Great Britain.*

[2] *RecycleLab Ltd., 101 New Cavendish Street, London, W1W 6XH, United Kingdom.*

[*] To whom correspondence should be addressed.
E-mail: c.banks@mmu.ac.uk; Tel: +44(0)1612471196

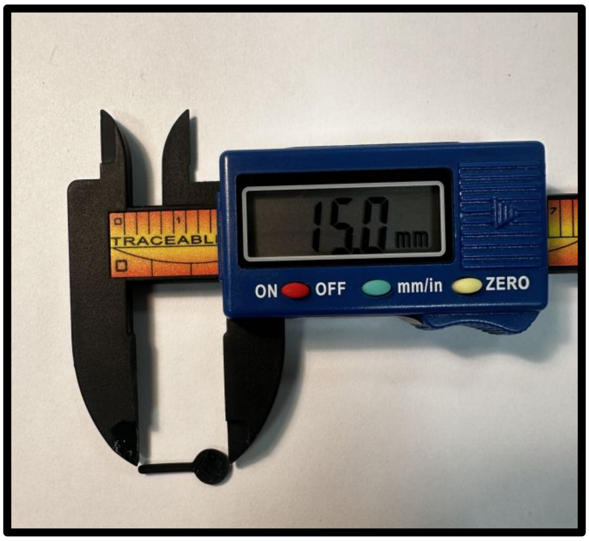

Figure S1. Image showing the length of the additively manufactured electrodes.

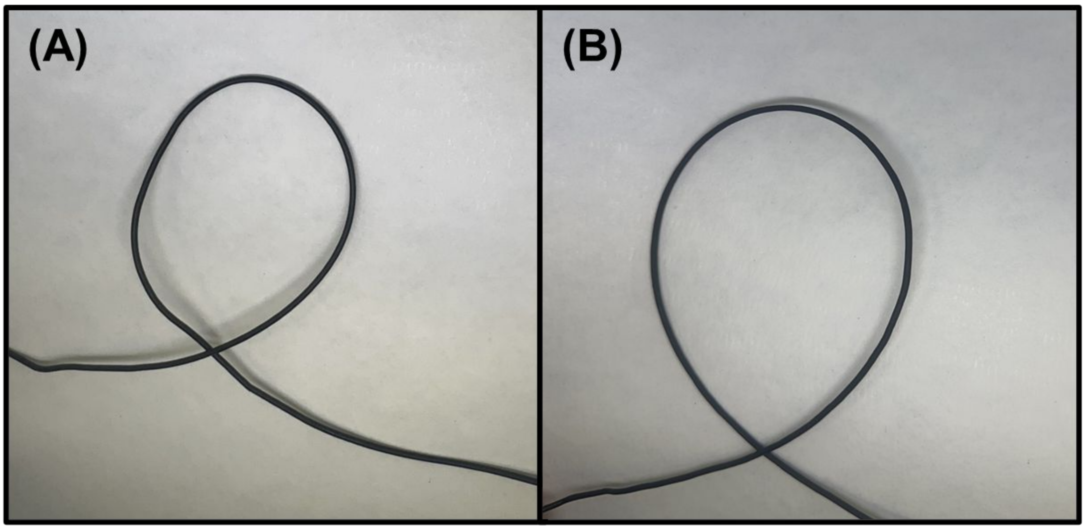

Figure S2. Images of conductive filaments of **A)** virgin **B)** hospital waste showing their flexibility.

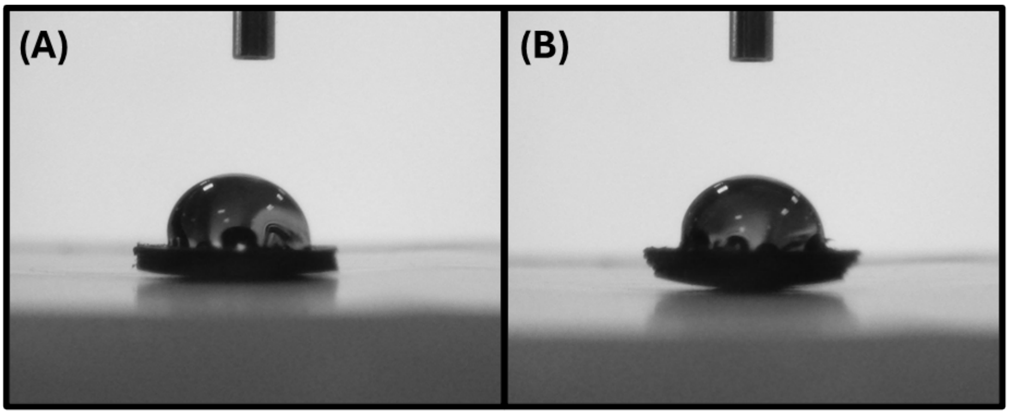

Figure S3. Images displaying contact angle experiment for **(A)** virgin **(B)** hospital waste electrode.

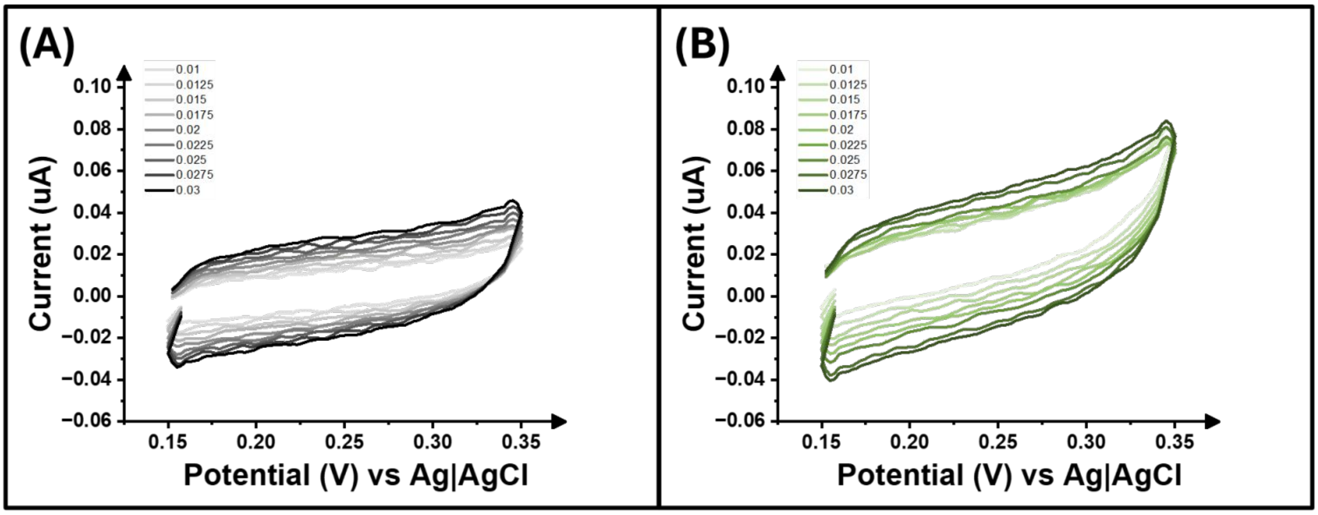

Figure S4. Cyclic voltammograms at varying scan rates (0.01 – 0.03 V s^{-1}) for **A)** virgin and **B)** hospital waste electrodes in 0.1 M KCl within non-Faradaic region.

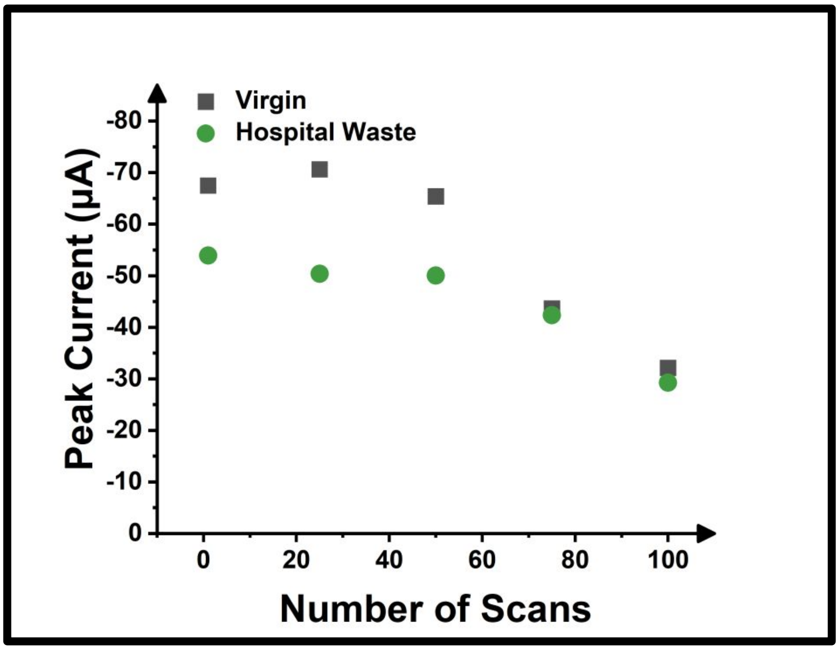

Figure S5. Analysis of cyclic voltammogram (50 mV s^{-1}) comparing virgin and hospital waste electrodes across 100 scans in $[Ru(NH_3)_6]^{3+}$ 1 mM in 0.1 M KCl.

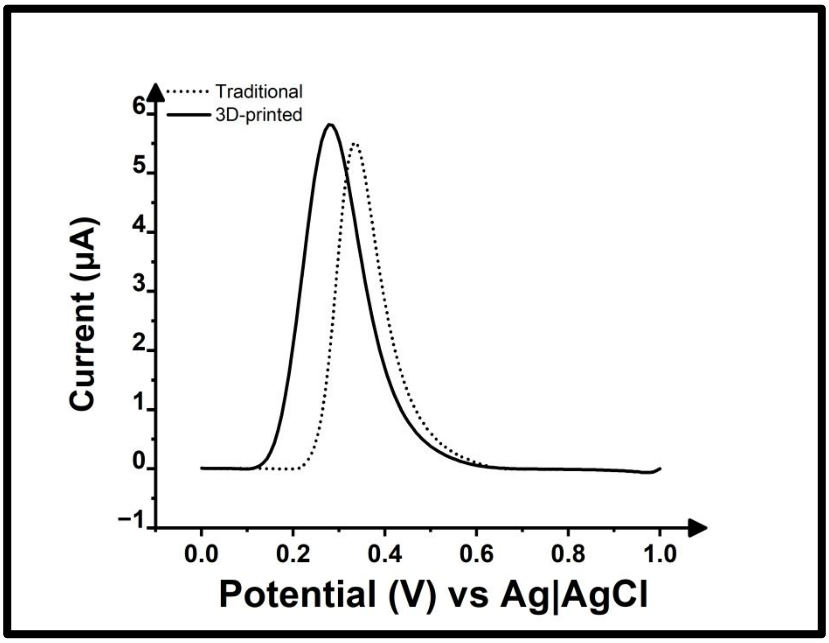

Figure S6. Differential pulse voltammogram for a virgin electrode in uric acid (60 μM in 0.01 M PBS) in a traditional set up (dotted) and a 3D printed setup (solid).

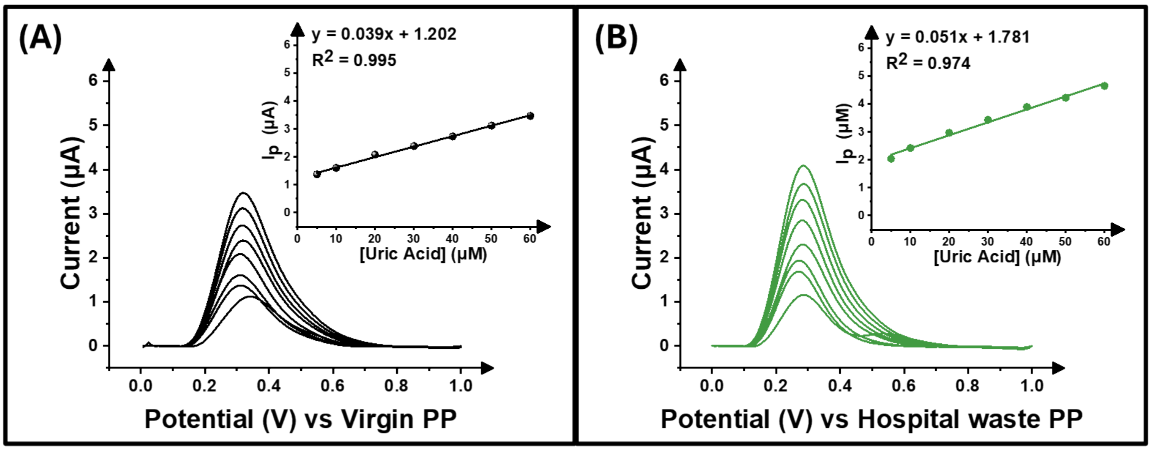

Figure S7. Differential pulse voltammogram for **(A)** virgin polypropylene **(B)** hospital waste electrodes for the recovery of 30 μM of uric acid in synthetic urine using the standard addition method with a concentration range of 5 μM – 60 μM in 0.01 M PBS. (inset = calibration plot)

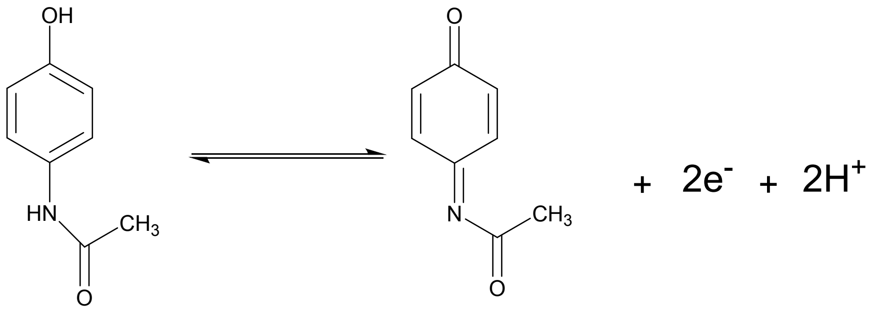

Mechanism S1. Proposed mechanism for the oxidation of acetaminophen.

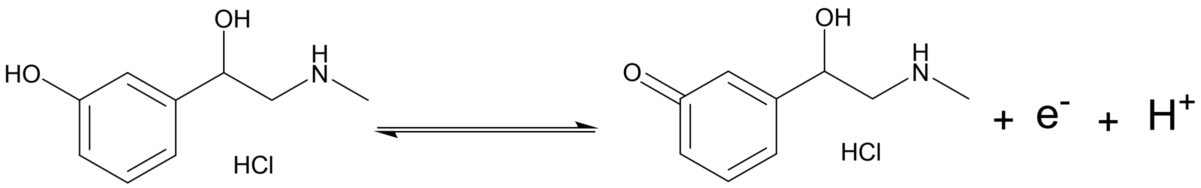

Mechanism S2. Proposed mechanism for the oxidation of phenylephrine.

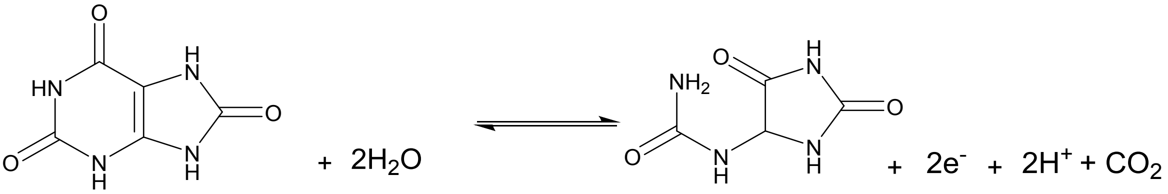

Mechanism S3. Proposed mechanism for the oxidation of uric acid.

Table S1. Comparison of results sensing acetaminophen.

Electrode Material	Electrode Modification	Electroanalytical Technique	Dynamic Range	Limit of Detection	Reference
GCE	MWCNTs- GNS	DPV	0.8 - 110	0.10	[1]
SPE	MXene	DPV	0.25 - 2000	0.04	[2]
GCE	SWCNTs–CHIT–RTIL	DPV	2 -200	0.11	[3]
GCE	CB-PAH	LSV	1 - 90	1.30	[4]
CPE	PR	DPV	0.7 - 100	0.53	[5]
GCE	AuNPs/poly(trisamine**)**	DPV	1.9 - 180	0.10	[6]
Recycled Polypropylene	CB	DPV	5 - 40	0.04	This work

Key: GCE: Glassy carbon electrode; SPE: Screen printed electrode; CPE: Carbon paste electrode; MWCNTs-GNS: MWCNT-graphene nanosheet nanocomposite; SWCNTs–CHIT–RTI: single walled carbon nanotube/chitosan/room temperature ionic liquid nanocomposite; CB-PAH: layering of carbon black/poly(Allylamine Hydrochloride); PR: Patton and Reeder's reagent; AuNPs/poly(trisamine): Gold-nanoparticles/poly(2-amino-2-hydroxymethyl-propane-1,3-diol) film; CB: Carbon black; DPV: differential pulse voltammetry; LSV: linear sweep voltammetry

Table S2. Comparison of results obtained from literature of sensing of phenylephrine.

Electrode Material	Electrode Modification	Electroanalytical Technique	Dynamic Range	Limit of Detection	Reference
GCE	$BiVO_4/ZrO_2$@graphene	DPV	0.009 - 120	6.9	[7]
CPE	MWCNT	CV	5 - 750	0.39	[8]
PLA	MWCNT/CB	DPV	5 - 60	0.38	[9]
Recycled Polypropylene	CB	DPV	5 - 40	0.03	This work

Key: GCE: Glassy carbon electrode; CPE: Carbon paste electrode; PLA: poly(lactic acid); $BiVO_4/ZrO_2$@graphene : bismuth vanadate/zirconium dioxide@graphene; MWCNT: multi walled carbon nanotubes; CB: Carbon black; DPV: differential pulse voltammetry; CV: cyclic voltammetry.

Table S3. Comparison of results obtained from literature of sensing of uric acid.

Electrode Material	Electrode Modification	Electroanalytical Technique	Dynamic Range (µM)	Limit of Detection (µM)	Reference
GCE	PCN/MWCNT	DPV	0.2 - 20	0.139	[10]
TPU	CB	DPV	10 - 60	0.53	[11]
GCE	PG	CV	6 – 1330	4.82	[12]
GCE	SWCNTs array	SWV	10 - 200	0.82	[13]
Recycled Polypropylene	CB	DPV	5 - 60	0.03	This work

Key: GCE: Glassy carbon electrode; CPE: Carbon paste electrode; TPU: Thermoplastic Polyurethane; PCN: porous g-C3N4; MWCNT: multi walled carbon nanotubes; SWCNT: single walled carbon nanotubes; PG: Pristine graphene; CB: Carbon black; DPV: differential pulse voltammetry; SWV: Square wave voltammetry; CV: Cyclic voltammetry.

References:

[1] M. Arvand, T.M. Gholizadeh, Simultaneous voltammetric determination of tyrosine and paracetamol using a carbon nanotube-graphene nanosheet nanocomposite modified electrode in human blood serum and pharmaceuticals, Colloids and Surfaces B: Biointerfaces, 103(2013) 84-93.
[2] Y. Zhang, X. Jiang, J. Zhang, H. Zhang, Y. Li, Simultaneous voltammetric determination of acetaminophen and isoniazid using MXene modified screen-printed electrode, Biosensors and Bioelectronics, 130(2019) 315-21.
[3] M. Afrasiabi, S. Kianipour, A. Babaei, A.A. Nasimi, M. Shabanian, A new sensor based on glassy carbon electrode modified with nanocomposite for simultaneous determination of acetaminophen, ascorbic acid and uric acid, Journal of Saudi Chemical Society, 20(2016) S480-S7.
[4] J. Scremin, G.J. Mattos, R.D. Crapnell, S.J. Rowley-Neale, C.E. Banks, E.R. Sartori, Glassy carbon electrode modified with layering of carbon black/poly (allylamine hydrochloride) composite for multianalyte determination, Electroanalysis, 33(2021) 526-36.
[5] T. Thomas, R.J. Mascarenhas, F. Cotta, K.S. Guha, B.K. Swamy, P. Martis, Z. Mekhalif, Poly (Patton and Reeder's reagent) modified carbon paste electrode for the sensitive detection of acetaminophen in biological fluid and pharmaceutical formulations, Colloids and Surfaces B: Biointerfaces, 101(2013) 91-6.
[6] M. Taei, G. Ramazani, Simultaneous determination of norepinephrine, acetaminophen and tyrosine by differential pulse voltammetry using Au-nanoparticles/poly (2-amino-2-hydroxymethyl-propane-1, 3-diol) film modified glassy carbon electrode, Colloids and Surfaces B: Biointerfaces, 123(2014) 23-32.
[7] E. Murugan, A. Poongan, A new sensitive electrochemical sensor based on BiVO4/ZrO2@graphene modified GCE for concurrent sensing of acetaminophen, phenylephrine hydrochloride and cytosine in medications and human serum samples, Diamond and Related Materials, 126(2022) 109117.
[8] Q. Zhou, H. Zhai, Y. Pan, Voltammetric determination of phenylephrine hydrochloride using a multi-walled carbon nanotube-modified carbon paste electrode, Royal Society Open Science, 5(2018) 181264.
[9] R.D. Crapnell, I.V.S. Arantes, J.R. Camargo, E. Bernalte, M.J. Whittingham, B.C. Janegitz, et al., Multi-walled carbon nanotubes/carbon black/rPLA for high-performance conductive additive manufacturing filament and the simultaneous detection of acetaminophen and phenylephrine, Microchimica Acta, 191(2024) 96.
[10] J. Lv, C. Li, S. Feng, S.-M. Chen, Y. Ding, C. Chen, et al., A novel electrochemical sensor for uric acid detection based on PCN/MWCNT, Ionics, 25(2019) 4437-45.
[11] A.C.M. Oliveira, E. Bernalte, R.D. Crapnell, M.J. Whittingham, R.A.A. Muñoz, C.E. Banks, Advances in additive manufacturing for flexible sensors: bespoke conductive TPU for multianalyte detection in biomedical applications, Applied Materials Today, 42(2025) 102597.
[12] S. Qi, B. Zhao, H. Tang, X. Jiang, Determination of ascorbic acid, dopamine, and uric acid by a novel electrochemical sensor based on pristine graphene, Electrochimica Acta, 161(2015) 395-402.
[13] Y. Yang, M. Li, Z. Zhu, A novel electrochemical sensor based on carbon nanotubes array for selective detection of dopamine or uric acid, Talanta, 201(2019) 295-300.
